# Long-Term Stability of Benefits of Cognitive Behavioral Therapy for Obsessive Compulsive Disorder Depends on Symptom Remission During Treatment

**DOI:** 10.32872/cpe.v2i1.2785

**Published:** 2020-03-31

**Authors:** Björn Elsner, Frieder Wolfsberger, Jessica Srp, Antonia Windsheimer, Laura Becker, Tanja Jacobi, Norbert Kathmann, Benedikt Reuter

**Affiliations:** a Department of Psychology, Humboldt-Universität zu Berlin, Berlin, Germany; Philipps-University of Marburg, Marburg, Germany

**Keywords:** obsessive-compulsive disorder, Y-BOCS, cut-off score, expert consensus, follow-up

## Abstract

**Background:**

Cognitive behavioral therapy (CBT) is an effective treatment for obsessive-compulsive disorder (OCD) and may afford stable long-term improvements. It is not clear, however, how stability or symptom recurrence can be predicted at the time of termination of CBT.

**Method:**

In a 1-year follow-up intention-to-treat study with 120 OCD patients receiving individual CBT at a university outpatient unit, we investigated the predictive value of international consensus criteria for response only (Y-BOCS score reduction by at least 35%) and remission status (Y-BOCS score ≤ 12). Secondly, we applied receiver-operating characteristic (ROC) curves in order to find an optimal cut-off score to classify for deterioration and for sustained gains.

**Results:**

Response only at post-treatment increased the likelihood of deterioration at follow-up compared to remission at an odds ratio of 8.8. Moreover, ROC curves indicated that a post-treatment score of ≥ 13 differentiated optimally between patients with and without symptom deterioration at follow-up assessment. The optimal cut-off score to classify for any sustained gains (response, remission, or both) at follow-up relative to baseline was 12. Importantly, previous findings of generally high long-term symptom stability after treatment in OCD could be replicated.

**Conclusion:**

The findings highlight the clinical importance of reaching remission during CBT, and suggest that a recently published expert consensus for defining remission has high utility.

## Abstract (highlights)

- A 1-year follow-up study with OCD patients having received a CBT trial was conducted.
- Achieving a Y-BOCS score ≤ 12 at termination of treatment decreases the risk of future deterioration.
- The study supports a rationale to treat OCD patients until reaching remission status.
- The study confirms the criterion for remission in OCD recently published as an expert consensus.

Cognitive behavioral therapy (CBT) is an effective treatment for obsessive-compulsive disorder (OCD). Its efficacy in randomized-controlled trials (RCT; Olatunji, Davis, Powers, & Smits, 2013; Öst, Havnen, Hansen, & Kvale, 2015) and its effectiveness in routine clinical practice (Hans & Hiller, 2013) have been confirmed in meta-analyses. According to follow-up data, treatment gains are largely maintained after treatment, but in randomized controlled trials, slight increases of average symptom scores from post-treatment to follow-up are observed at group level (Olatunji et al., 2013; Öst et al., 2015). However, follow-up data from routine care are still rare, especially for individual outpatient therapy (Cabedo, Carrió, & Belloch, 2018; Hans & Hiller, 2013; Hansen, Kvale, Hagen, Havnen, & Öst, 2019).

The Yale-Brown Obsessive-Compulsive Scale (Y-BOCS) interview (Goodman et al., 1989a; Goodman et al., 1989b) has been established as the "gold standard" to measure OCD symptom severity, and is commonly used as a primary outcome measure (Öst et al., 2015). Effect sizes based on Y-BOCS group mean scores are therefore useful for comparisons between studies and interventions, and allow observing within-group changes. However, group mean scores do not reflect individual improvement (Hiller, Schindler, Andor, & Rist, 2011; Jacobson, Follette, & Revenstorf, 1984), which is especially important in research on routine clinical practice. In order to address this issue, Jacobson and Truax (1991) proposed a definition of clinically significant improvement by combining statistically significant changes in individual symptoms (Reliable Change Index, RCI) with subclinical symptom levels. This makes it possible to determine individual response (without remission), remission, and deterioration. Since clinically significant change depends on the reliability of the measure and the variance in the relevant population, cut-off scores for remission varied between 7 and 16 across published studies (Öst et al., 2015). Subsequently, Mataix-Cols et al. (2016) published an international expert consensus on change assessment in OCD, in which treatment response was defined as a reduction in Y-BOCS scores by at least 35% and an improvement score of 1 (“very much improved”) or 2 (“much improved”) on the Clinical Global Impression scale (CGI, Guy, 1976). For remission, a Y-BOCS score of < 13 and CGI severity ratings of 1 (“normal, not at all ill”) or 2 (“borderline mentally ill”) must be achieved. These criteria have been adopted in recent research (Hansen et al., 2019) and may prove influential for future clinical decisions in OCD treatment. Yet, it remains unclear whether these consensus criteria have clinical utility and are able to predict individual long-term stability.

Prediction of post-treatment response and remission on the basis of pre-treatment Y-BOCS scores has been investigated by means of signal detection analyses (Farris, McLean, Van Meter, Simpson, & Foa, 2013). Criteria evaluation for predicting outcome at follow-up, however, is missing. Prospective studies on depressive disorder and social phobia suggest that incomplete remission at post-treatment predicts relapse at follow-up (Judd et al., 1998; Paykel et al., 1995; Van Ameringen et al., 2003). In line with these results, two studies with OCD patients have shown that “partial remission” compared to “full remission” at the end of treatment predicts relapse during follow-up periods of one to five years (Braga, Cordioli, Niederauer, & Manfro, 2005; Braga, Manfro, Niederauer, & Cordioli, 2010; Eisen et al., 2013). One of these studies (Eisen et al., 2013), however, did not use Y-BOCS scores for the evaluation of clinical status. In the other, full remission required a Y-BOCS score of < 8 (Braga et al., 2005; Braga et al., 2010), which is much stricter than the consensus Y-BOCS cut-off score for remission (≤ 12). It is therefore unclear whether the protective effect of “full remission” can also be found when applying the less strictly defined remission criterion. Prediction of long-term stability is of major importance for clinical practice, because under routine conditions the criterion for terminating individual psychotherapy is often not specified in advance. Treatment may be continued until a “good enough level” (GEL) is achieved (Barkham et al., 2006; Falkenström, Josefsson, Berggren, & Holmqvist, 2016), which is often defined subjectively by patient and therapist. Clinical decisions, however, should also be informed by empirical research. In addition to testing the predictive value of categorical variables such as remission or response, it is also worthwhile to determine the exact post-treatment Y-BOCS scores that separate patients with stable treatment gains from those with loss of gains in the follow-up period, or patients with long-term improvements in relation to pre-treatment levels from those without such improvements. If good prediction is possible on the basis of a single, widely-used and easy-to-apply instrument, the cut-off scores can inform clinical decisions on whether to terminate or to continue CBT.

In the present study, we conducted a 1-year follow-up assessment in a relatively large sample of OCD patients, who had received individual CBT under routine conditions of the German health care system. Our main goals were: 1.) testing whether patients achieving the consensus Y-BOCS cut-off score for remission at post-treatment are less likely to experience significant symptom increase at follow-up compared to unremitted responders, 2.) determining a post-treatment Y-BOCS cut-off score that differentiates optimally between patients who deteriorate from post-treatment to follow-up and those whose initial improvement remains stable, and 3.) determining a post-treatment Y-BOCS cut-off score that predicts for any sustained gains (response, remission, or both) at follow-up. A secondary aim was to provide further data for evaluations of average and individual symptom changes from pre- and post-treatment to follow-up in a treatment setting typical for routine care in many countries.

## Method

### Participants

Study participants had terminated individual cognitive behavioral therapy (CBT) at a university outpatient unit (Hochschulambulanz für Psychotherapie und Psychodiagnostik der Humboldt-Universität zu Berlin) between December 2013 and May 2017. Referrals to the outpatient unit were made according to routine clinical care procedures. Patients who prematurely discontinued CBT (non-completers) were not excluded and the last observation was carried forward to estimate post-treatment scores (interim-assessments were done every 20 sessions). General study inclusion criteria were: primary diagnosis of OCD, age between 18 and 70 years, and a minimum pre-treatment Y-BOCS total score of 16. Due to general admission policies of the outpatient unit, patients with comorbid psychotic disorders, borderline personality disorder, or substance dependency (life time) were not referred. Three patients were excluded from analysis due to missing Y-BOCS-data at both pre- and post-treatment. During the study period, a total of 207 patients fulfilled the inclusion criteria and were contacted by telephone for follow-up assessments. Among these, 51 (24.6%) patients could not be reached and 36 (17.4%) declined to participate. 120 patients participated in the phone interview (58.0% of the total sample), and 96 of them completed additional online questionnaires (46.4% of the total sample).

Participants (*n* = 120, 75 female, 104 therapy completers) and non-participants (*n* = 87, 49 female, 70 therapy completers) in the follow-up interview did not differ significantly in terms of gender (*p* = .392), therapy completer status (*p* = .252), or other demographic and clinical variables (see Table 1). For both participants and non-participants, the most common comorbid mental disorders were present or remitted depressive disorders and anxiety disorders. Twenty-four patients of the total sample suffered from personality disorders (see Table 2). 73 patients took psychotropic medications at admission (35.3%), 55 at post-treatment (26.6%); the most common medications were selective serotonin reuptake inhibitors (SSRIs) and other antidepressants. The study protocol was approved by the local review board of Humboldt-Universität zu Berlin (protocol number 2016-33) and met the ethical standards of the revised Declaration of Helsinki. All participants provided written informed consent.

**Table 1:** Demographic and Clinical Variables of Participants and Non-Participants in Follow-up Assessments

Variable	Participants Assessment t_FU_		Non-Participants Assessment t_FU_		*t*-test for independent samples		
	*n*	*M* (*SD*)	*n*	*M* (*SD*)	*df*	*t*	*p*
Age	120	32.3 (9.5)	87	31.5 (9.9)	181.0	0.59	.558
Age of symptom onset	109	17.1 (8.8)	81	17.1 (7.6)	183.2	0.02	.986
Age of disorder onset	110	23.1 (9.6)	78	22.4 (8.5)	117.3	0.58	.558
Duration of therapy (hours)	119	41.0 (17.6)	87	42.3 (20.7)	167.4	-0.45	.653
Socio-economic status	112	9.6 (3.7)	79	9.2 (4.2)	154.2	0.77	.443
GAF t__pre__	118	55.8 (10.1)	86	53.3 (11.2)	171.0	1.66	.099
Y-BOCS t_pre_	120	23.3 (4.6)	87	24.4 (4.7)	182.0	-1.74	.083
Y-BOCS t__post__	120	11.9 (7.3)	87	13.7 (7.7)	178.8	-1.69	.092
OCI-R t__pre__	118	27.1 (13.0)	87	29.4 (12.4)	190.1	-1.31	.193
OCI-R t__post__	120	14.4 (12.0)	85	17.7 (13.5)	167.2	-1.78	.078
BDI-II t__pre__	119	18.9 (11.2)	87	20.4 (10.8)	188.8	-0.97	.336
BDI-II t__post__	120	9.8 (8.7)	84	10.9 (11.3)	148.6	-0.78	.438
BSI-GSI t__pre__	119	0.98 (0.5)	87	1.01 (0.6)	178.0	-0.37	.712
BSI-GSI t__post__	120	0.60 (0.5)	85	0.70 (0.6)	163.2	-1.17	.245

*Note*. GAF = Global Assessment of Functioning; Y-BOCS = Yale-Brown Obsessive-Compulsive Scale interview score; OCI-R = Obsessive Compulsive Inventory - Revised; BDI-II = Beck Depression Inventory II; BSI-GSI = Global Severity Index of the Brief Symptom Inventory; pre = pre-treatment; post = post-treatment; FU = 1-year follow-up.

**Table 2:** Most Common Comorbid Mental Disorders and Medication Status at t_pre_ and t_post_.

Condition	Participants Assessment t_FU_		Non-Participants Assessment t_FU_	
	*n*	%	*n*	%
≥ 1 comorbid mental disorder	76	63.3	51	58.6
present depressive disorder	40	33.3	29	33.3
remitted depressive disorder	28	23.3	20	23.0
any anxiety disorder	41	34.2	15	17.2
Personality disorder	12	10.0	12	13.8
Psychotropic medications t_pre_	45	37.5	28	32.2
Psychotropic medications t_post_	35	29.2	20	23.0

*Note.* pre = pre-treatment; post = post-treatment; FU = 1-year follow-up.

### Treatment

CBT was administered by nineteen licensed psychotherapists, who had completed at least three years of training in CBT. Treatments were bound to the general conditions for psychotherapy in the public German health care system. The legal framework allowed up to 66.7 hours (80 units of 50 minutes each) per treatment. Therapists were instructed to apply CBT including exposure and response prevention (ERP) according to the national guideline for evidence-based treatment (Hohagen, Wahl-Kordon, Lotz-Rambaldi, & Muche-Borowski, 2014). Adherence was not formally controlled and treatment was not manualized, but therapists received weekly supervision by one of four experienced CBT therapists. Therapy sessions usually lasted 50 minutes and took place once or twice weekly, yet therapists were free to adjust session length when implementing exposure and to reduce session frequency at the end of treatment. Treatment was terminated by consensus of patient and therapist based on clinical criteria. Patients who abandoned treatment without the approval of their therapist were classified as non-completers.

### Assessment

One-year follow-up status of patients (t_FU_) was assessed by telephone-based interviews and internet-based self-report questionnaires. Analyses also included data from routine assessments at admission (t_pre_) and termination of therapy (t_post_), and for non-completers, from interim-assessments.

Telephone interviews were conducted by trained master level psychology students, who were supervised by an experienced psychotherapist (B.R.). Interviews included the German version of the Y-BOCS interview to assess OCD symptom severity (Goodman et al., 1989a; Goodman et al., 1989b; Hand & Büttner-Westphal, 1991). Internet-based assessments included the Obsessive Compulsive Inventory - Revised (OCI-R, Foa et al., 2002) as a secondary outcome measure of OCD symptoms, the Beck Depression Inventory II (BDI-II, Beck, Steer, & Brown, 1996) to measure current depression, and the Brief Symptom Inventory (BSI, Derogatis & Melisaratos, 1983) to assess general psychological distress using its Global Severity Index.

Routine assessments at admission (t_pre_) included the German version of the Structured Clinical Interviews for DSM-IV mental disorders and personality disorders (SCID-I, SCID-II, First, Gibbon, Spitzer, Williams, & Benjamin, 1997; First, Spitzer, Gibbon, & Williams, 1995), and a socio-economic status scale (Lampert & Kroll, 2009). In order to assess symptom course, Y-BOCS interview, OCI-R, BDI-II, BSI, the clinical global impression scale (CGI, Guy, 1976) and the global assessment of functioning (GAF, Jones, Thornicroft, Coffey, & Dunn, 1995) were administered before the first and after the final therapy session. Interim assessments were conducted every 20 sessions and used to estimate post-treatment data for non-completers without post-treatment assessments (*n* = 10; last-observation-carried-forward method). Interim assessments were also used to estimate post-treatment scores for four therapy completers with missing data. All clinical interviews at admission and post-treatment were conducted by trained clinical psychologists.

### Data Analysis

We analyzed data using R version 1.0.44. Participants and non-participants were compared using independent two sample *t*-tests (two-sided). Fisher’s exact test was applied to compare nominal data. Effect sizes were calculated using Cohen’s *d* with pooled standard deviations. Changes over time were compared with paired *t*-tests (two-sided). We used the expert consensus criteria (Mataix-Cols et al., 2016) for Y-BOCS scores to define remission (total score ≤ 12), response (reduction ≥ 35%), and non-response (reduction < 35%), but did not apply the CGI improvement scale (see also Hansen et al., 2019). We used the Reliable Change Index (RCI, Jacobson et al., 1984) to define statistically meaningful deterioration (e.g. Bablas, Yap, Cunnington, Swieca, & Greenwood, 2016; Han, Geffen, Browning, Kenardy, & Geffen, 2011; Kraus, Castonguay, Boswell, Nordberg, & Hayes, 2011). To calculate the RCI, an internal consistency of α = .79 (Moritz et al., 2002) was used as the reliability of the Y-BOCS. Stability was defined as the absence of significant deterioration. Logistic regression analysis was used to contrast response without remission (response only) and remission at post-treatment to predict deterioration at follow-up. As we were interested in stability after initial improvement, patients with no response during treatment were not considered in this analysis. Additionally, we applied receiver-operating characteristic (ROC) curves using R package OptimalCutpoints (López-Ratón, Rodríguez-Álvarez, Cadarso-Suárez, & Gude-Sampedro, 2014) in order to find the best post-treatment Y-BOCS score classifying for deterioration versus stability at follow-up. ROC curves were also used to find the optimal post-treatment cut-off score classifying for sustained gains (response, remission, or both; *n* = 77) at follow-up. The score that reached a maximum Youden index (J = Sensitivity + Specificity - 1; Youden, 1950) was considered as optimal cut-off.

## Results

### Average Symptom Change

**Figure 1:**
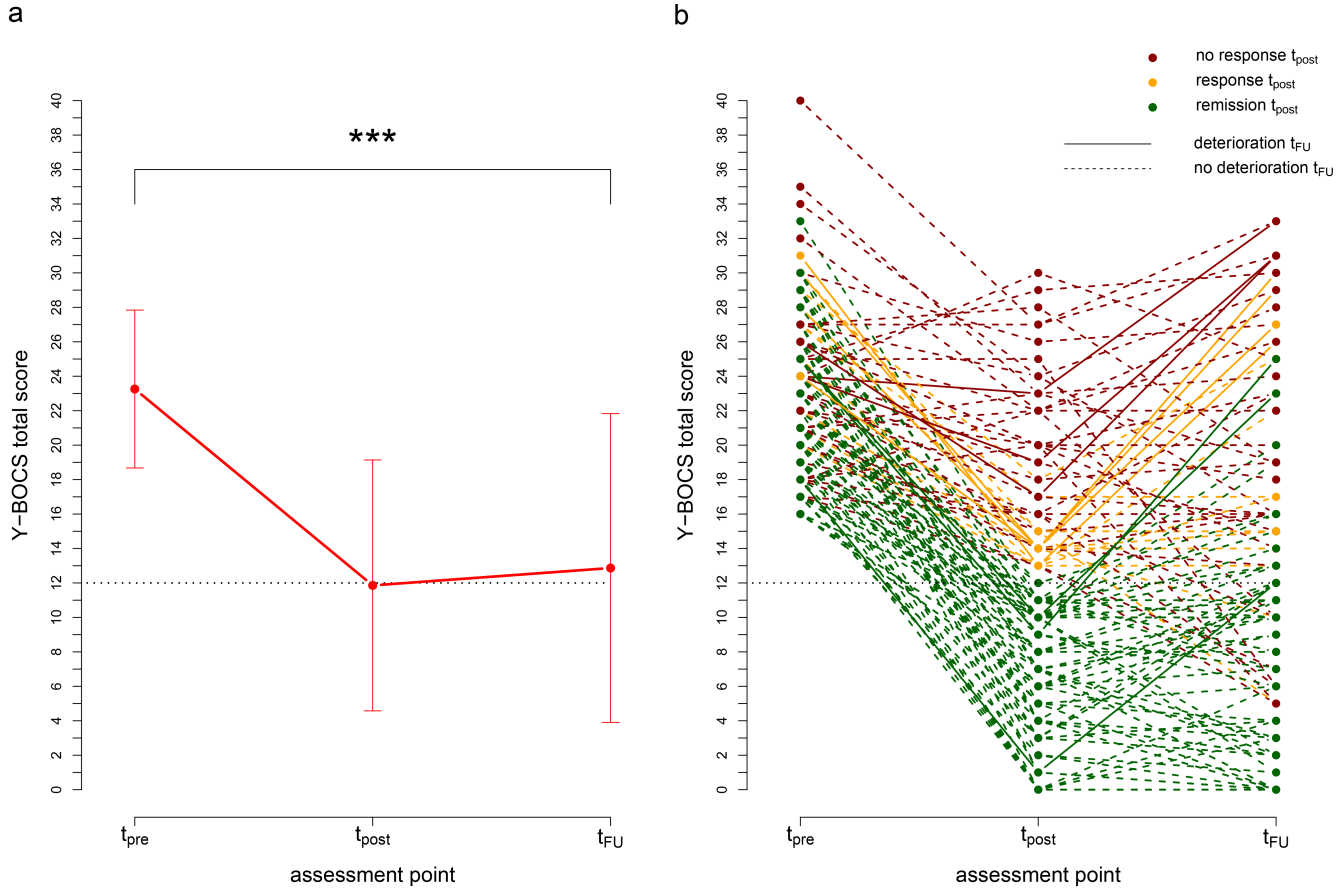
Average and Individual Symptom Change *Note.* a) Mean Y-BOCS total scores at pre-treatment (t_pre_), post-treatment (t_post_) and follow-up (t_FU_). b) Individual remission, response only and non-response at post-treatment (according to the expert consensus) and significant deterioration (according to Reliable Change Index) from post-treatment to follow-up. Error bars indicate standard deviations. ***p < .001.

On group level, the Y-BOCS score decreased significantly from pre-treatment to post-treatment, *t*(119) = 17.23, *p* < .001, with a mean reduction of 11.4 points and a large effect size of Cohen’s *d* = 1.87 (Figure 1a, Table 3). Symptom severity was also significantly reduced from pre-treatment to one-year follow-up, *t*(119) = 13.75, *p* < .001, *d* = 1.46. The increase of the mean Y-BOCS score from post-treatment to follow-up was small, but close to significance, *t*(119) = -1.79; *p* = .076, *d* = -0.12 (see Figure 1a).

Similarly, secondary outcome parameters showed significant reductions from pre-treatment to post-treatment (OCI-R: *t*(234) = 7.82; *p* < .001, *d* = 1.01; BDI-II: *t*(222.99) = 7.03; *p* < .001, *d* = 0.91 and BSI-GSI: *t*(236.01) = 5.62; *p* < .001, *d* = 0.73), and from pre-treatment to follow-up (OCI-R: *t*(208.32) = 8.40, *p* < .001, *d* = 1.14; BDI-II: *t*(196.88) = 4.15; *p* < .001, *d* = 0.57; and BSI-GSI: *t*(207.95) = 5.21; *p* < .001, *d* = 0.71). No significant change from post-treatment to follow-up was observed for OCI-R, *t*(205.13) = 0.83, *p* = .409, *d* = 0.11; for BDI-II, *t*(168.30) = -1.71, *p* = .089, *d* = -0.24; and for BSI-GSI, *t*(204.06) = -0.12, *p* = .903, *d* = -0.02; (see Table 3).

**Table 3:** Mean Differences and Effect Sizes From Pre-Treatment and Post-Treatment to Follow-up

Measure	*n*_FU_	*M*_FU_ (*SD*)	*M*_post_ (*SD*)	*d*_post-FU_	*M*_pre_ (*SD*)	*d*_pre-FU_
Y-BOCS	120	12.9 (9.0)	11.9 (7.3)	-0.12	23.3 (4.6)	1.46
OCI-R	94	13.1 (11.3)	14.4 (12.0)	0.11	27.1 (13.0)	1.14
BDI-II	96	12.3 (12.0)	9.8 (8.7)	-0.24	18.9 (11.2)	0.57
BSI-GSI	96	0.61 (0.5)	0.60 (0.5)	-0.02	0.98 (0.5)	0.71

*Note.* Y-BOCS = Yale-Brown Obsessive-Compulsive Scale interview score; OCI-R = Obsessive Compulsive Inventory - Revised; BDI-II = Beck Depression Inventory II; BSI-GSI = Global Severity Index of the Brief Symptom Inventory; pre = pre-treatment; post = post-treatment; FU = 1-year follow-up.

### Individual Improvement

The course of symptoms from pre-treatment to post-treatment and follow-up was heterogeneous across patients (Figure 1b). Table 4 displays the numbers of patients with non-response, response without remission (response only), and remission at post-treatment and follow-up. Adopting the RCI for deterioration, Table 5 shows the numbers of participants with Y-BOCS score stability and deterioration at follow-up broken down by their outcome category at post-treatment. The relationship between outcome category (remission, response only, non-response) at post-treatment and stability at follow-up is illustrated in Figure 1b.

**Table 4:** Number of Non-Responders, Responders Without Remission and Remitters for Post-Treatment and Follow-up

Outcome category at t_post_	Outcome category at 1-year follow-up (t_FU_)			
	No response	Response only	Remission	Σ_post_
No response	27	3	7	37 (30.8%)
Response only	8	4	2	14 (11.7%)
Remission	8	9	52	69 (57.5%)
Σ_FU_	43 (35.8%)	16 (13.3%)	61 (50.8%)	120 (100%)

*Note.* Response only = Response without remission; post = post-treatment; FU = 1-year follow-up.

**Table 5:** Change During Follow-up: Number of Stable and Deteriorated Participants at Follow-up Broken Down by Their Outcome Category at Post-Treatment

Outcome category at t_post_	Change during 1-year follow-up (t_FU_)		
	Stability	Deterioration	Σ_post_
No response	34	3	37 (30.8%)
Response only	10	4	14 (11,7%)
Remission	66	3	69 (57.5%)
Σ_FU_	110 (91.7%)	10 (8.3%)	120 (100%)

*Note.* Response only = Response without remission; post = post-treatment; FU = 1-year follow-up.

### Prediction of Long-Term Outcomes

Compared to remission, response only significantly predicted deterioration at follow-up (*B* = 2.17, *SE* = 0.84, χ^2^(1) = 6.58, *p* = .010, Odds Ratio (OR) = 8.8, CI = 1.71 - 50.65, Wald χ^2^ = 6.77, *p* = .009). Nagelkerke's *R*-squared of this model was .174 (Hosmer-Lemeshow *R^2^* = .137, Cox-Snell *R^2^* = .076). The inclusion of Y-BOCS scores at pre-treatment as predictor did not improve the model significantly, *B* = 0.13 (*SE* = 0.11), *p* = .235. Initial Y-BOCS scores did not predict deterioration, OR = 1.1 (CI = 0.92 - 1.44, Wald χ^2^ = 1.41, *p* = .245).

### Cut-off Scores

**Figure 2:**
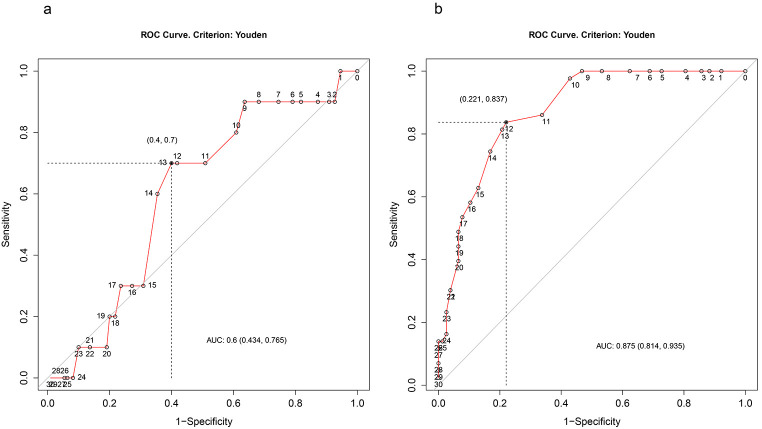
Cut-off Points on the Y-BOCS *Note.* Receiver-operating characteristic (ROC) curves with optimal cut-off points on the Y-BOCS at post-treatment to classify a) for deterioration (vs. stability) at follow-up and b) for sustained gains (response, remission, or both) at follow-up. AUC = Area under the ROC curve.

The Y-BOCS score at post-treatment that best predicted significant deterioration versus stability was 13 (sensitivity = .70; specificity = .60), indicating that participants with a score higher than or equal to 13 were more likely deteriorated at follow-up (see Figure 2a). Interestingly, the optimal cut-off score predicting sustained gains (relative to baseline) was 12 (sensitivity = .83; specificity = .78), suggesting that a Y-BOCS score of 12 or less at the time of treatment termination predicts sustained benefits at one year follow-up (see Figure 2b).

### Medication and Subsequent Outpatient Therapy

Sixty-seven patients were free of psychotropic medications from post-treatment to follow-up. Twenty patients discontinued medications after post-treatment, but seven of them were again medicated at follow-up. Thirty patients were medicated continuously from post-treatment to follow-up. For three patients, data about medication at follow-up was missing. Most common were SSRIs (*n* = 33). A significant association between medication status (no medication, discontinued, discontinued and medicated again, continuously medicated) and outcome category at follow-up was observed (*p* = .015), with higher remission rates for medication-free patients and discontinuers (61.2% and 69.2%) than for continuously medicated patients (26.7%). No significant association could be observed for medication status and deterioration (*p* = .402) at follow-up assessment.

Eighteen patients sought additional outpatient therapy of more than five sessions after post-treatment. Subsequent therapy was neither correlated with outcome category at post-treatment (*p* = .067), nor at follow-up assessment (*p* = .086), but at both assessment points, patients without remission sought additional therapy more frequently than remitters on a trend level.

## Discussion

The present study aimed to examine whether remission status and symptom levels at post-treatment are predictive for long-term stability of improvements after cognitive behavioral therapy for OCD. In addition, we intended to evaluate the general effectiveness of individual cognitive behavioral therapy in a sample of 120 patients by conducting a follow-up assessment one year after termination of treatment in routine clinical practice.

Applying the recently published Y-BOCS consensus criteria (Mataix-Cols et al., 2016) to classify patients as non-responders, responders, or remitters showed that response only at post-treatment was associated with a significantly higher likelihood for deterioration. Among the patients who benefited from CBT, those who achieved remission by the end of treatment had a considerably higher chance of maintaining initial improvement. Given the fact that stability and deterioration were defined by absence or presence of reliable changes (RCI), the criterion variable was not confounded with the consensus criteria. While similar findings have been shown in previous studies, these applied different remission criteria (Braga et al., 2005; Braga et al., 2010; Eisen et al., 2013). To our knowledge, the present findings are the first to show the predictive value of the consensually recommended Y-BOCS cut-off score, and thus confirm its validity in terms of long-term stability.

Considering that different cut-off scores have proven to predict long-term stability, we sought to determine a Y-BOCS score at post-treatment that best predicts deterioration versus stability one year later. Receiver-operating characteristic (ROC) curves pointed to a cut-off point of ≥ 13 for classifying for future deterioration. As stability until follow-up may not be sufficient to assume long-term improvement, we finally determined a cut-off score to classify for sustained benefits at follow-up relative to pre-treatment. The resulting cut-off score of ≤ 12 implies that patients with a Y-BOCS score of twelve or lower at post-treatment are likely to show long-term therapy benefits compared to patients with higher scores. Notably, the identified critical symptom levels are almost identical to the proposed expert consensus cut-off score for remission.

These findings highlight the utility of a Y-BOCS cut-off score of ≤ 12 for defining remission status at post-treatment and add to previous evidence that subthreshold symptom severity protects patients with mental disorders from later deterioration (Braga et al., 2005; Braga et al., 2010; Judd et al., 1998; Paykel et al., 1995; Van Ameringen et al., 2003).

The results have implications for both etiological models and clinical practice. Different etiological models (Kalanthroff, Abramovitch, Steinman, Abramowitz, & Simpson, 2016; Robbins, Gillan, Smith, de Wit, & Ersche, 2012; Salkovskis, 1999) emphasize that compulsions contribute to the maintenance or worsening of symptoms. A reduction of symptom severity below a critical threshold may therefore weaken these dynamics. In clinical practice, the question of how to proceed if patients achieve response but not remission during the scheduled duration of psychotherapy is central. Ethical considerations may support continuation of treatment until remission is achieved. However, while there is research on treatment of non-responders to pharmacological therapy (Albert et al., 2018; Denys, van Megen, van der Wee, & Westenberg, 2004; Pallanti, Hollander, & Goodman, 2004), there is little data on the treatment of patients who failed to reach remission status during CBT.

As we observed large effect sizes for pre-post (*d* = 1.87) and pre-FU (*d* = 1.46) periods, we were able to confirm previous findings of long-term effectiveness of individual outpatient CBT in OCD (Cabedo et al., 2018; Hans & Hiller, 2013; Hansen et al., 2019). Although our results suggest that reduced symptom levels are maintained from post-treatment to follow-up, we did observe a slight, non-significant increase in symptoms. Recurrence of OCD symptoms after treatment termination has been found in previous follow-up studies (Anderson & Rees, 2007; Barrett, Healy-Farrell, & March, 2004; Bolton & Perrin, 2008), yet not consistently (Rufer et al., 2005). The slight increase in the present study may be explained by inferior long-term symptom stability of the small group of patients that achieved response without remission: while most patients who remitted (75.4%) or did not respond (73.0%) at post-treatment remained in the same outcome category at follow-up, only 28.6% of responders remained in this category one year later. Very few patients with response (without remission) at post-treatment achieve remission one year later (14.3%), which illustrates again that response only at post-treatment indicates insufficient treatment.

One limitation of the present study stems from the treatment setting under routine conditions. Particularly, treatment did not follow a specific manual and therapy adherence was not controlled. The mean duration of therapy was longer than in most RCTs. Note, however, that “high intensity interventions” with more than 30 therapist-hours per patient have been found to yield superior effect sizes for treatment outcome compared to low and medium intensity (National Collaborating Centre for Mental Health, 2006). In the present study, the relatively long duration results from individual treatment planning, consideration of comorbid disorders, and termination of treatment on the basis of a consensual decision of patient and therapist. The duration is comparable to the average duration of outpatient psychotherapy in the public health care system in Germany (Lutz, Wittmann, Böhnke, Rubel, & Steffanowski, 2012). Thus, our data derive from treatment conditions that are typical for the German and similar health care systems and may provide high ecological validity.

Sample size constitutes another limitation, as, at post-treatment, we observed only 14 patients in the category of response without remission, and only ten participants with deterioration at follow-up. Although, considering the large number of remitted patients that indicates an overall very successful treatment, larger sample sizes would increase the statistical power of predictions of critical subgroups. Future follow-up studies should also address life events, other therapies, and medications that may influence symptom stability. Furthermore, longer follow-up intervals might enable us to make conclusions about predictors of long-term treatment benefits.

In summary, the present results suggest that the symptom level reached when terminating treatment is critical for the future course of illness. A post-treatment Y-BOCS score < 13 optimally predicts higher individual likelihood for stability one year later. This cut-off almost perfectly fits with the expert consensus criterion for remission of OCD. Thus, such a remission criterion may be a useful instrument in aiding decision making in routine clinical practice, in particular for terminating or continuing treatment.
